# Supplementary material for: A quantitative microbial risk assessment for touchscreen user interfaces using an asymmetric transfer gradient transmission mode
Source: PLoS One. 2022 Mar 25;17(3):e0265565. doi: 10.1371/journal.pone.0265565 (PMC8956170; doi:10.1371/journal.pone.0265565)
Supplement: S1 Appendix — (PDF) [file pone.0265565.s001.pdf]

Appendix

A Markov Chains & Queueing Networks

A first-order Markov Chain can be used to control the flow of people between (for example) 3 zones / locations A,B and C (illustrated in Fig 2) At each time step in the simulation, a random number of individuals are extracted from the departure queues at each location at a rate of $\lambda_{Z_A}, \lambda_{Z_B}$ and λ_{Z_C}. Where these individuals go next is determined by the *state transition matrix* of the Markov Chain. Note, each column of the matrix should sum to 1.

$$\begin{pmatrix} P(A) \\ P(B) \\ P(C) \end{pmatrix}_n = \begin{pmatrix} P(A|A) & P(A|B) & P(A|C) \\ P(B|A) & P(B|B) & P(B|C) \\ P(C|A) & P(C|B) & P(C|C) \end{pmatrix} \begin{pmatrix} P(A) \\ P(B) \\ P(C) \end{pmatrix}_{n-1} \tag{A.1}$$

Consider a person initially at A: Then generate a uniform random variable $u \sim U(0,1)$. *If* $u \le P(A|A)$ that person stays at A (actually they rejoin to the 'arrival' queue of A). *Else, if* $u \le P(A|A) + P(B|A)$ they go to B. Otherwise, they go to C i.e. $u \le P(A|A) + P(B|A) + P(C|A) = 1$ since we've exhausted the possible destinations.

We can add further control by giving each individual a maximum number of 'jumps' (or transitions) between zones; once reached the individual can be directed to a 'dummy' location to await the end of the simulation for post processing of the results.

B Confidence Intervals

With respect to Alice and Bob, the outcome of any one simulation realisation is either to be 'infected' or not. Thus, the output is a series of n Bernoulli random trials. For large n we may use the Gaussian approximation for the estimation of the confidence interval:

$$CI_{(95\%)} = p \pm 1.96\sqrt{\frac{p(1-p)}{n}} \tag{B.1}$$

where $p \in [0,1]$ is the mean infection rate averaged over n trials. Note: the maximum value that $\sqrt{p(1-p)}$ can take is 0.5. Therefore, by selecting $n = 40000$ we can be 95% confident that our infection rate is within at most $\pm 0.49\%$ about our measured value.

C Exponential Moving Average Cumulative Sum

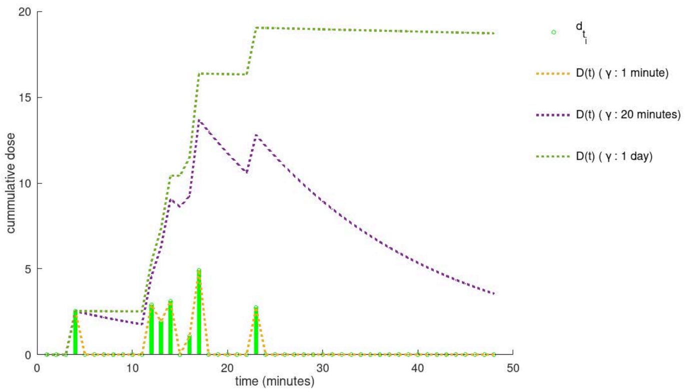

Fig 8. Cummulative dose as a function of inoculation period γ. Green lines (d_{t_i}) represent simulated random inoculation events (or instantaneous dose). For large γ (1 day) these events are not 'forgotten'; they accumulate as a standard cumulative summation. For $\gamma = 20$ minutes, the effective dose dies-off over time, reducing the likelihood of infection. This is due to pathogen decay in mucus and immune system activity. For extremely small γ, only the instantaneous dose at the time of the inoculation event plays a role in infection risk.

D Default Parameters

Table 1. Simulation default parameters.

Symbol	Description	Value
-	(initial) Bioburden of A	10^5
ID_{50}	Infectious Dose (50% probability)	10^2
d_s	Pathogen survival on surface $\% \times hr^{-1}$	0.8
d_f	Pathogen survival on skin (finger) $\% \times hr^{-1}$	0.01
-	Decontamination rate (hr^{-1}) at A and B	0
-	Pathogen Survival After Cleaning‡	$\sim Lognormal(\mu = -3.0187, \sigma = 0.614)$
-	Face touch events (per hour)*	$\sim Pois(15)$
-	Personal-touch events (per hour)*	$\sim Pois(204)$
α_{face}	Deposit rate (finger to face)	$\sim f_{tn}(0.35, 0.1, 0, 0.6)$
α_{pt}	Deposit rate (personal-touch events e.g. clothing)	$\sim f_{tn}(0.05, 0.1, 0, 0.6)$
γ	Inoculation period (minutes)	20
α	Deposit rate (tui)	$\sim f_{tn}(0.05, 0.3, 0, 1)$
β	Pick-up rate (tui)	$\sim f_{tn}(0.27, 0.3, 0, 1)$
n_t	TUI interface touch rate	$\sim f(12, 3.5, 8, 40)$
λ_{TUI}	TUI throughput (people/min)	$\sim Pois(0.5)$
λ_A, λ_B	Departure rate from A and B (people/min)†	$\sim Pois(0.5)$

Default simulation parameters. Further details are displayed in Fig 3. Note: $f_{tn}(\mu, \sigma, a, b)$ refers to the truncated normal distribution with domain [a,b].

*these values are divided by 10 to account for 1 finger involved in TUI use.

‡This equates to (1 - cleaning efficiency) where cleaning efficiency is 94.1% with CI_{95} = [71.4 , 98.8] (see [44])

†Everyone enters the simualtion from an initial population pool at $\sim Pois(0.25)$ i.e. once every 4 minutes. Thus we would expect the simulation to take approximately 2 hrs. Alice and Bob are programmed to enter the simualtion $\sim Pois(0.0167)$ i.e. once per hour; they arrive (on average) as if from the middle of the initial population queue.
