# Supplementary material for: A quantitative microbial risk assessment for touchscreen user interfaces using an asymmetric transfer gradient transmission mode
Source: PLoS One. 2022 Mar 25;17(3):e0265565. doi: 10.1371/journal.pone.0265565 (PMC8956170; doi:10.1371/journal.pone.0265565)
Supplement: Attachment — Submitted filename: Response to Reviewers.docx [file pone.0265565.s003.docx]

**Response to reviewers**

Reviewer Comments –> Author’s Response

Reviewer #1:

*The paper PONE-D-21-23569 entitled " A quantitative microbial risk assessment for touchscreen user interfaces using an asymmetric transfer gradient transmission mode" discusses an important issue which is the quantitative microbial risk assessment. In particular, the author wished to know if touchscreens can transmit enough pathogens to a user to cause an infection.

My major concerns regarding the study are the lack of validation of the results obtained. Although it would be difficult to determine the number of infections, it should be at least possible to determine if the results presented in Fig. 6 are close to reality or how they compare with other studies with infection risk in the order of 1-2.5%. Models are important to simulate scenarios but it should be possible to assess how close to reality are the results of each scenario.*

*One thing that is not clear to the reader is if the risk determined is high or not. How can 1-2.5% infection risk be considered? How can this be compared to other situations?*

A discussion of risk assessment is presented in a new section (Discusssion: Risk Assessment). It includes additional references to similar fomite studies (one simulated, one based on a real outbreak). Infection risk varies considerably. By comparison, the 2-3% risk from Fig.6 is not exceptional. However, the section also discusses the concept of risk and why data regarding fomite related (real-world) outbreaks is harder to come by. Essentially, the paragraph attempts to explain why it may (or may not) be considered ‘high’ risk depending on certain other variables e.g. the severity of the contracted illness. Ultimately, risk is highly subjective.

The author also discusses the use of disinfectants and UV light to decrease the risk of transmission. Could this favour the risk of selecting more resistant strains?

Indeed. A mention of this phenomenon (and reference specific to UV use) was added to the end of section (Discussion: Decontamination)

Specific comments are listed below:

- The Methods section should allow the repetition of the study by other colleagues. Information about the software and language used to make the simulations/model should be given here. The author added information about the simulator framework only at the end of the paper, but more information is required in the Methods section.

Further information about the code is provided in a new section in Methods ( *Simulation Code*). In a previous model and publication, much of the method section was devoted to code. While this was very informative to a computer science crowd, it did obscure ‘the big picture’ of how the algorithm works.

I have provided links to instructions in a readme file + example scripts. Results are in .csv format and can be easily displayed in any spreadsheet program or similar.

- The discussion section also contains results that should be presented in the results section (e.g. contamination levels, dose response, etc). A general discussion about what the impact of the different assumptions and choices made is lacking. The author presents the discussion in different sub-sections and states the assumptions made but does not provide what would be the result if another choice was made. With the computational model, the impact of some assumptions could be tested to provide support to the decisions made.

A paragraph was added to *Simulation Parameter Sensitivity* section within Results that summarizes the main result i.e. the ~3% risk. This is later re-iterated and discussed in the Discussion section.

The suitability of the scenario tested was already discussed in *Suitability of simulated scenario* of the Discussion. Further discussion was added (Discussion: *Risk Assessment* and *Model Limitations*) to discuss results and some of the model’s limitations. There are an infinite number of scenarios one could choose to simulate. The aim of this paper was to highlight the model’s ability to incorporate re-deposition of microbes onto surfaces. A simple scenario was chosen to keep the interpretation brief, straightforward and provide generalised insight into fomite/TUI mediated disease transmission.

- A general conclusion to the questions presented as aims of the study is not provided.

Discussion: *Risk Assessment* discusses the results specifically in terms of the QMRA. The risk e.g. 3% is discussed in context. In terms of which pathogen types are likely to infect someone using a TUI; it seems imprudent to list any specifically owing to the in-exactitudes of measuring bacterial/viral contamination. It can be stated that large initial bioburdens of pathogens with high infectiousness and long half-life are the key factors in transmission (which is probably not surprising). An interesting discussion point: The pathogen survival (half-life) needs to be taken into context of the rate of TUI use. An extremely long survival on surfaces is irrelevant if TUIs are used at high rates, as pathogen removal will be dominated by pickup followed by personal-touching.

- English should be checked throughout the text. Examples of phrases that should be improved follow:
+ “A more concerning source of contamination if from a user(s) with unwashed hands, particularly after toilet use.”

A restructure of the sentence was implemented (see edited text)
+ “All of these methods suffer when administered inappropriately or by insufficiently
trained staff.” (Methods do not suffer..:)

‘The effectiveness of all of these methods is reduced when…. ‘
+ “Each simulation results is averaged (…)”
result (not results)

Furthermore, the author should not use the word “we” in the text. Usually scientific studies are written without referring to who did the work. In this particular case, only one author is listed and the use of "we" should be avoided.

All instances of ‘we’ (where applicable) were eliminated and replaced with an appropriate sentence re-structure.

- “Another important assumption in this simulation is that people will interact with a TUI using just one finger e.g. index finger of the dominant hand.” – This may be true for older people but younger people use more fingers in touchscreens. In the discussion, decisions such as this should be discussed and their impact assessed.

Discussion: *Model Limitations* now discusses this issue. Essentially, it would be simple enough programmatically to simply ‘add’ more ‘fingers’. However, it leads to further complications. The current model assumes on dominant finger and therefore the entire surface of the TUI essentially becomes homogenised. Adding more fingers would require to then break up the surface into sub-areas where each finger is likely to touch (e.g. during a typing gesture). There can also be cross-contamination between fingers of the same person. This would be something to look into possibly in future work, particularly if one was to adapt the model to simulate a very specific touch interface.

It may be possible to ‘hack’ this behaviour into the existing framework by describing these separate regions of the same screen as separate locations (each with its own Markov chain, touch rate parameters etc..). But I believe this would deserve a separate experiment and write-up.

As stated in *Model Limitations*, the current model ‘averages out’ these details and gives more generalised results; this is not necessarily a bad thing as it allows us to see the bigger picture of how the TUI and network of individuals interact. Much like an SIR model teaches us about pandemics despite wrapping all of human/pathogen behaviour in a single R value.

Reviewer #2 (specific comments)

1. Introduction
   Pg.1 L7-8: The sentence is awkward, suggest connecting sentence to this study.
    Sentence restructured as follows: “Thus, the aim of this work was to carry out a QMRA using computer simulation methods, in an attempt to answer these questions.”

Pg.2 L44: Change “are deposited onto as surface”... to “are deposited onto a surface”... Pg.2 L47-49: Change the word “simulates” to “simulate”.
Well spotted and apologies for the silly mistake.

Pg.3 L76-77. Suggest author to change sentence to refer to the study not to the author (40). Removed ‘by the author’

2. Methods
   Pg.3 L92: Fig. 1. Well described

Thank you.

Pg.3 L94: Suggest author add references to Table 1 on a right-side column.

Certainly, hopefully the final typesetter can help with this?

Pg.4 L99: Fig. 2. Well described

Thank you.

Pg.4 L123: Fig. 3. Clarify the units on the y-axis, should it be log10 as in Fig. 5?

The units are linear for figure 3. This better represented the different decay rate and equilibrium points reached at different parameter settings. Figure 5 required a log10 scale to accentuate the behaviour (particularly in B).

Pg.6 L175: Suggest the author describes the simulation software coding language C and provide benefit in discussion section.

New mention at end of Methods section (*Simulation Code*). A further discussion of the simulator framework can be found in Discussion (*Simulator Framework*).

3. Results:
   Pg.6 L202: The ~3% infection risk of public touchscreens mentioned in the abstract is not mentioned in the results nor in the discussion.
4. Discussion:
   Pg.8 L224: The ~3% infection risk of public touchscreens mentioned in the abstract is not mentioned in the discussion section.

See response to Reviewer #1: sections have been added to Results and Discussion to deal with the 3% result along with other important discussion points.
