# Supplementary material for: A quantitative microbial risk assessment for touchscreen user interfaces using an asymmetric transfer gradient transmission mode
Source: PLoS One. 2022 Mar 25;17(3):e0265565. doi: 10.1371/journal.pone.0265565 (PMC8956170; doi:10.1371/journal.pone.0265565)
Supplement: Attachment — Submitted filename: Reviewer comments-PONE-D-21-23569.pdf [file pone.0265565.s002.pdf]

The study titled: “A quantitative microbial risk assessment for touchscreen user interfaces using an asymmetric transfer gradient transmission mode” and conducted by the author provides a rationale that gives a risk assessment of TUI use from a generic pathogen type under plausible circumstances. Comparing risk from primary and second-hand exposure of 32 individuals going to two locations (A and B) using a queueing network was an important and distinctive property of the asymmetric gradient transmission model to determine the risk to two individuals (Alice and Bob). The model allowed for estimation of an individual's finger bioburden over time and subsequent cross-contamination of surfaces. The overall risk of public touchscreens was ~3%. The research topic studied here is definitely a salient one, and manuscript is generally well written.

The author conducted a well-organized study, developing a simulation scenario with multiple input parameters and modeling the scenario in the coding language C. Overall, rationale of the study, analyzed sample size, and methodological approach are reasonable. The authors’ major findings presented here that parameter sensitivity on infection risks are based on n=40,000 realizations with 95% CI making the QMRA scenario scientifically sound and should be of interest to the journal. The manuscript is considered for publication with minor revisions.

Specific comments:

1. The authors conducted an excellent study and provided an appropriate amount of literature review on the topic.
2. The parameters tested in the QMRA i.e., queueing network, transfer rates-asymmetric gradient model, dynamic equilibrium of surface bioburden and the additional sources of pathogen removal provides a thorough approach validating the simulation.
3. Introducing the coding language C in the methods section and taking a step-by-step approach of explaining how the simulation was performed would be helpful for readers to utilize the simulation model. Excellent idea to provide the link to the code used in the study and the appendix information.

Minor modifications (Pg. Page L: Line):

Abstract:

Well written

1. Introduction

   Pg.1 L7-8: The sentence is awkward, suggest connecting sentence to this study.

   Pg.2 L44: Change "are deposited onto as surface"… to "are deposited onto a surface"…

   Pg.2 L47-49: Change the word "simulates" to "simulate".

   Pg.3 L76-77. Suggest author to change sentence to refer to the study not to the author (40).

2. Methods

   Pg.3 L92: Fig. 1. Well described

   Pg.3 L94: Suggest author add references to Table 1 on a right-side column.

   Pg.4 L99: Fig. 2. Well described

   Pg.4 L123: Fig. 3. Clarify the units on the y-axis, should it be log10 as in Fig. 5?

   Pg.6 L175: Suggest the author describes the simulation software coding language C and provide benefit in discussion section.

3. Results:

   Pg.6 L202: The ~3% infection risk of public touchscreens mentioned in the abstract is not mentioned in the results nor in the discussion.

4. Discussion:

   Pg.8 L224: The ~3% infection risk of public touchscreens mentioned in the abstract is not mentioned in the discussion section.

References:

Well written
